# Comparative efficacy of novel biomaterials versus traditional materials in the treatment of dental and pulpal diseases: a systematic review and meta-analysis

**DOI:** 10.3389/fmed.2026.1762823

**Published:** 2026-02-17

**Authors:** Qing Sheng, Guangwei Fan, Weiqing Lu, Linran Yao, Bochong Li, Yijun Qian, Danping Zheng, Shi Zhang, Ruyi Zhu, Yanyan Hu

**Affiliations:** 1 Department of Endodontics, Dental & Ophthalmic Clinic of Putuo District, Shanghai, China; 2 Department of Periodontist, Dental & Ophthalmic Clinic of Putuo District, Shanghai, China; 3 Department of Prosthodontics, Dental & Ophthalmic Clinic of Putuo District, Shanghai, China

**Keywords:** apical sealing, bioceramic sealer, meta-analysis, postoperative pain, root canal treatment, single-cone technique

## Abstract

**Aim:**

This systematic review and meta-analysis aimed to compare the efficacy of novel bioceramic sealers versus traditional epoxy resin-based sealers (AH Plus) used with the single-cone technique in root canal treatment, focusing on postoperative pain, apical sealing, overfilling, retreatment difficulty, and clinical success.

**Methods:**

Following PRISMA guidelines, a comprehensive search was conducted across PubMed, Embase, Cochrane CENTRAL, Web of Science, CNKI, and Wanfang databases from January 2014 to December 2024. Randomized controlled trials (RCTs) and cohort studies comparing bioceramic sealers (e.g., iRoot SP, Bio-C Sealer) to AH Plus were included. Two reviewers independently extracted data and assessed quality using Cochrane RoB 2.0 and Newcastle-Ottawa tools. Meta-analyses were performed using RevMan 5.4, employing fixed- or random-effects models based on heterogeneity (*I*^2^).

**Results:**

Fifteen studies (11 RCTs, 4 cohorts; 1,528 teeth) were included. Bioceramic sealers significantly reduced postoperative pain (OR = 0.48, 95% CI: 0.35–0.66, *p* < 0.001; *I*^2^ = 22%) and improved apical sealing (SMD = −1.35, 95% CI: −1.78 to −0.92, *p* < 0.001; *I*^2^ = 71%). However, they increased overfilling risk (OR = 1.85, 95% CI: 1.25–2.74, *p* = 0.002; *I*^2^ = 18%) and retreatment difficulty (SMD = 0.88, 95% CI: 0.25–1.51, *p* = 0.006; *I*^2^ = 45%). Mid-term clinical success rates showed no significant difference (OR = 1.12, 95% CI: 0.85–1.47, *p* = 0.41; *I*^2^ = 15%).

**Conclusion:**

Bioceramic sealers outperform AH Plus in reducing pain and enhancing sealing but carry higher overfilling and retreatment challenges. Mid-term success rates are comparable. Clinicians should prioritize bioceramics for cases demanding superior sealing and comfort while employing careful technique to mitigate risks. Long-term studies are needed to validate durability and retreatment protocols.

**Systematic review registration:**

PROSPERO, identifier (CRD420261285302).

## 1 Introduction

Dental pulp diseases are among the most common clinical conditions in dentistry. The core of their treatment lies in the complete removal of infectious sources and achieving lasting sealing of the root canal system (1, 2). As a key intervention for endodontic and periapical diseases, the long-term success of root canal treatment largely depends on the performance of root canal filling materials and the precision of clinical procedures (3, 4). For decades, traditional epoxy resin-based sealers (such as AH Plus) have been widely used due to their high mechanical strength and stable physicochemical properties (5, 6). However, these materials also have significant limitations: slight shrinkage may occur during polymerization, leading to microleakage; chemical components in their composition may cause tissue irritation; furthermore, their performance in terms of biocompatibility and promoting peripheral tissue regeneration is relatively limited (7–9).

In recent years, with advancements in biomaterials science, novel bioceramic-based root canal sealers have been gradually applied in clinical practice and have demonstrated significant advantages (5, 10). These materials primarily consist of calcium silicate, with representative products including internationally widely used iRoot SP and domestically produced C-Root SP, among others (11, 12). Upon contact with tissue fluids, they undergo a hydration reaction, generating nano-hydroxyapatite, which forms a chemical bond with the dentinal wall, greatly enhancing apical sealing ability (13, 14). Studies have shown that such materials not only exhibit excellent biocompatibility and antimicrobial activity but also promote osteogenic differentiation and cementum regeneration, demonstrating great potential in improving the healing environment of periapical tissues (15, 16).

With the evolution of obturation strategies, the hydraulic single-cone technique used with bioceramic sealers has gained popularity because of its procedural simplicity and reduced technique sensitivity, potentially improving efficiency in complex anatomies such as curved or C-shaped canal systems. Nevertheless, the relatively high flow and larger volume of sealer used in single-cone fillings may increase the risk of sealer extrusion/overfilling. In addition, the strong dentin bonding and mineral infiltration associated with premixed bioceramic sealers may compromise retreatability, making complete removal more challenging during non-surgical retreatment (17, 18).

Although several studies have explored the application effects of bioceramic sealers in root canal treatment, there is still a lack of systematic integration of large-sample, multicenter clinical data. Particularly regarding direct comparisons with traditional materials, key indicators such as postoperative pain control, microleakage performance, long-term sealing stability, and retreatability feasibility remain controversial or inadequately supported by evidence. Furthermore, previous literature has mostly focused on discussions of individual indicators, lacking a multidimensional analysis from the perspective of comprehensive clinical efficacy (19).

Therefore, this study aims to comprehensively compare multiple outcome indicators of novel bioceramic sealers versus traditional epoxy resin-based sealers in root canal treatment using the single-cone technique through systematic review and meta-analysis. These indicators include the incidence of postoperative pain, apical sealing ability, depth of dentinal tubule penetration, occurrence of overfilling, retreatment difficulty, and success rate, thereby providing evidence-based references for clinical material selection and operational standards.

## 2 Materials and methods

### 2.1 Literature search strategy

To ensure comprehensiveness and reproducibility, this study followed the PRISMA guidelines for systematic retrieval and developed a structured search strategy. Electronic databases searched included PubMed, Embase, the Cochrane Central Register of Controlled Trials (CENTRAL), the Web of Science Core Collection, as well as CNKI (China National Knowledge Infrastructure) and Wanfang Data Knowledge Service Platform. The search timeframe was set from January 2014 to December 2024 to cover relevant clinical studies from the past decade.

The search combined Medical Subject Headings (MeSH) and free-text terms. Core English keywords included: “Bioceramic sealer,” “Calcium silicate-based sealer”, “iRoot SP”, “Endosequence sealer”, “C-Root SP”, “Single-cone technique”, “Single cone obturation”, “Root canal filling”, “AH Plus”, “Epoxy resin”, “Outcome”, “Pain”, “Apical sealing”, “Microleakage”, “Obturation quality”, and “Retreatment.” As an example, the PubMed search strategy was as follows:

(((“bioceramic sealer”[Title/Abstract]) OR (“calcium silicate sealer”[Title/Abstract])) OR (“iRoot SP”[Title/Abstract])) AND ((“single-cone”[Title/Abstract]) OR (“root canal obturation”[Title/Abstract])) AND ((“AH Plus”[Title/Abstract]) OR (“epoxy resin”[Title/Abstract])).

Additionally, to minimize omissions, we manually screened the reference lists of included studies and tracked original articles cited in relevant reviews. All retrieved records were imported into EndNote X9 for management, and duplicates were removed both automatically and manually.

### 2.2 Inclusion and exclusion criteria

This study applied explicit and stringent inclusion (PICOS) and exclusion criteria.

Inclusion criteria:

1. Study design: Only randomized controlled trials (RCTs) and prospective or retrospective cohort studies published in peer-reviewed journals.
2. Participants: Patients with fully developed roots in single or multi-rooted permanent teeth undergoing root canal treatment, diagnosed with pulp necrosis or apical periodontitis.
3. Intervention: The experimental group used single-cone obturation with a novel bioceramic root canal sealer (including but not limited to iRoot SP, Endosequence BC Sealer, C-Root SP, Bio-C Sealer).
4. Comparison: The control group used lateral compaction or single-cone technique with traditional epoxy resin-based sealers (primarily AH Plus).
5. Outcomes: Studies must report at least one of the following primary or secondary outcomes.

Primary outcomes:

- Postoperative pain incidence (commonly defined as VAS score ≥4 at 24, 48, or 72 h post-operation).
- Apical sealing ability (microleakage value measured by dye penetration, fluid filtration, or glucose leakage, in μm or μg/mL).
- Overfilling rate (visible sealer extrusion beyond the apical foramen on periapical radiographs or CBCT).

Secondary outcomes:

- Dentinal tubule penetration depth (measured under microscopy).
- Retreatment difficulty (measured by retreatment time, percentage of remaining material, or number of instruments required).
- Short- to mid-term (6–24-month) clinical/radiographic success rate.

In this review, postoperative pain, overfilling, retreatment difficulty, and clinical/radiographic success were considered clinical outcomes, whereas apical sealing ability (microleakage) and dentinal tubule penetration were regarded as surrogate or laboratory-based outcomes.

Exclusion criteria:

Non-English publications; animal studies, *in vitro* studies, case reports, expert commentaries, conference abstracts; studies with incomplete data, unavailable full texts, or unreachable authors for data acquisition; studies involving primary teeth, teeth with open apices, or retreatment cases (unless initial treatment data were separately available); studies where mixed sealer types or obturation techniques prevented isolation of data for the target comparison.

### 2.3 Data extraction and quality assessment

Data extraction was conducted independently by two trained researchers (A and B). A predefined standardized form was used, and any disagreements were resolved through discussion or by a third researcher (C). Extracted information included:

1. Basic study characteristics: first author, publication year, country, and study design.
2. Participant characteristics: sample size (number of teeth), patient age, gender, and tooth position.
3. Intervention and comparison details: sealer brand and type, core gutta-percha type, and obturation technique specifics.
4. Outcome data: number of patients with pain at each time point, mean and standard deviation of microleakage, counts of overfilling, retreatment time, number of clinical successes, etc. For continuous variables, mean, standard deviation (SD), and sample size (*n*) were extracted; for dichotomous variables, event counts and total sample sizes were recorded.

Methodological quality was also assessed independently by two researchers. For RCTs, the Cochrane Risk of Bias tool (RoB 2.0) was used, evaluating domains including randomization process, deviations from intended interventions, missing outcome data, outcome measurement, and selective reporting. For cohort studies, the Newcastle-Ottawa Scale (NOS) was applied, with stars assigned across three domains: selection of participants, comparability of groups, and outcome assessment. A total score ≥7 stars indicated high quality.

### 2.4 Statistical analysis

All statistical analyses were performed using Review Manager (RevMan) software (version 5.4). For dichotomous variables (e.g., postoperative pain incidence, overfilling rate), the odds ratio (OR) with 95% confidence interval (CI) was used as the effect measure. For continuous variables (e.g., microleakage value, retreatment time), the mean difference (MD) was applied if the unit of measurement was identical; otherwise, the standardized mean difference (SMD) with 95% CI was used.

Heterogeneity among studies was assessed using the Chi-square test and *I*^2^ statistic. If *p* ≥ 0.1 and *I*^2^ ≤ 50%, heterogeneity was considered acceptable, and a fixed-effect model was used for meta-analysis. If *p* < 0.1 or *I*^2^ > 50%, significant heterogeneity was assumed, and a random-effects model was employed. Possible sources of heterogeneity (e.g., sealer brand, tooth type, follow-up time) were explored via subgroup or sensitivity analyses.

Potential publication bias was evaluated using funnel plots supplemented by Egger’s test. Sensitivity analysis was conducted for primary outcomes by sequentially excluding individual studies to test the robustness of the pooled results. The significance level for all statistical tests was set at *α* = 0.05.

## 3 Results

### 3.1 Study inclusion

**Figure 1:**
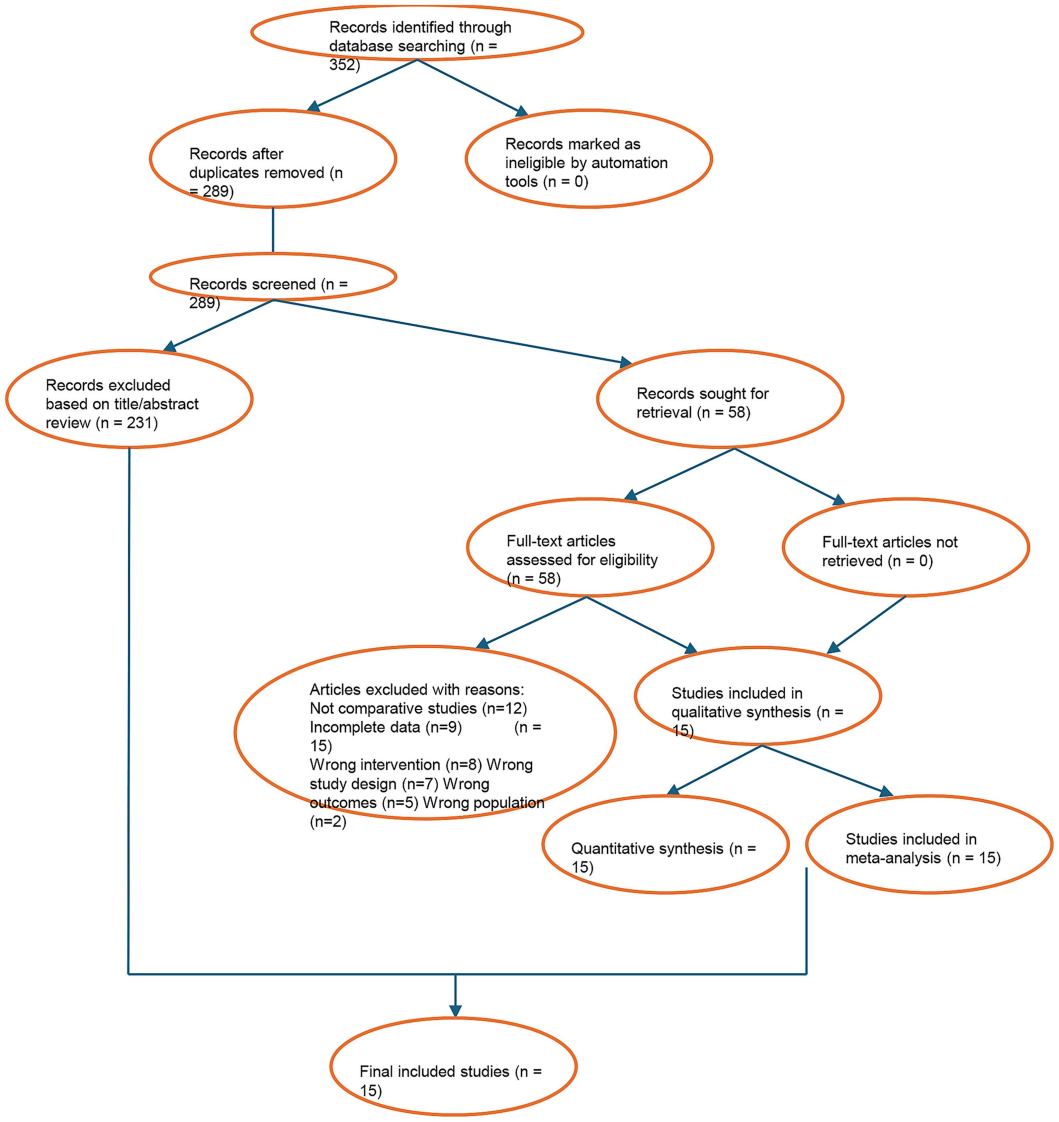
PRISMA flow diagram of the study selection process.

A total of 352 relevant records were initially identified. After removing duplicates using EndNote, 289 articles remained. Following title and abstract screening, 231 clearly irrelevant studies (e.g., *in vitro* research, animal studies, or off-topic publications) were excluded, leaving 58 articles for full-text assessment. Ultimately, 15 clinical studies meeting the eligibility criteria were included, comprising 11 randomized controlled trials (RCTs) and 4 prospective cohort studies, with a total of 1,528 treated teeth (768 in the bioceramic group and 760 in the traditional material group) (Supplementary Table 1). Figure 1 (flow diagram) illustrates the detailed study selection process and reasons for exclusion, with the most common reasons being “non-comparative study” (*n* = 12) and “unable to extract or combine data” (*n* = 9). The basic characteristics of the included studies are summarized in Supplementary Table 1. The publication years ranged from 2019 to 2023, and sample sizes varied from 68 to 150 teeth. All studies explicitly reported the brands of bioceramic sealers used (iRoot SP, *n* = 5; Bio-C Sealer, *n* = 4; C-Root SP, *n* = 3; Total Fill BC Sealer, *n* = 1; others, *n* = 2) and the control material (AH Plus, *n* = 15).

### 3.2 Primary outcome measures

#### 3.2.1 Postoperative pain

**Figure 2:**
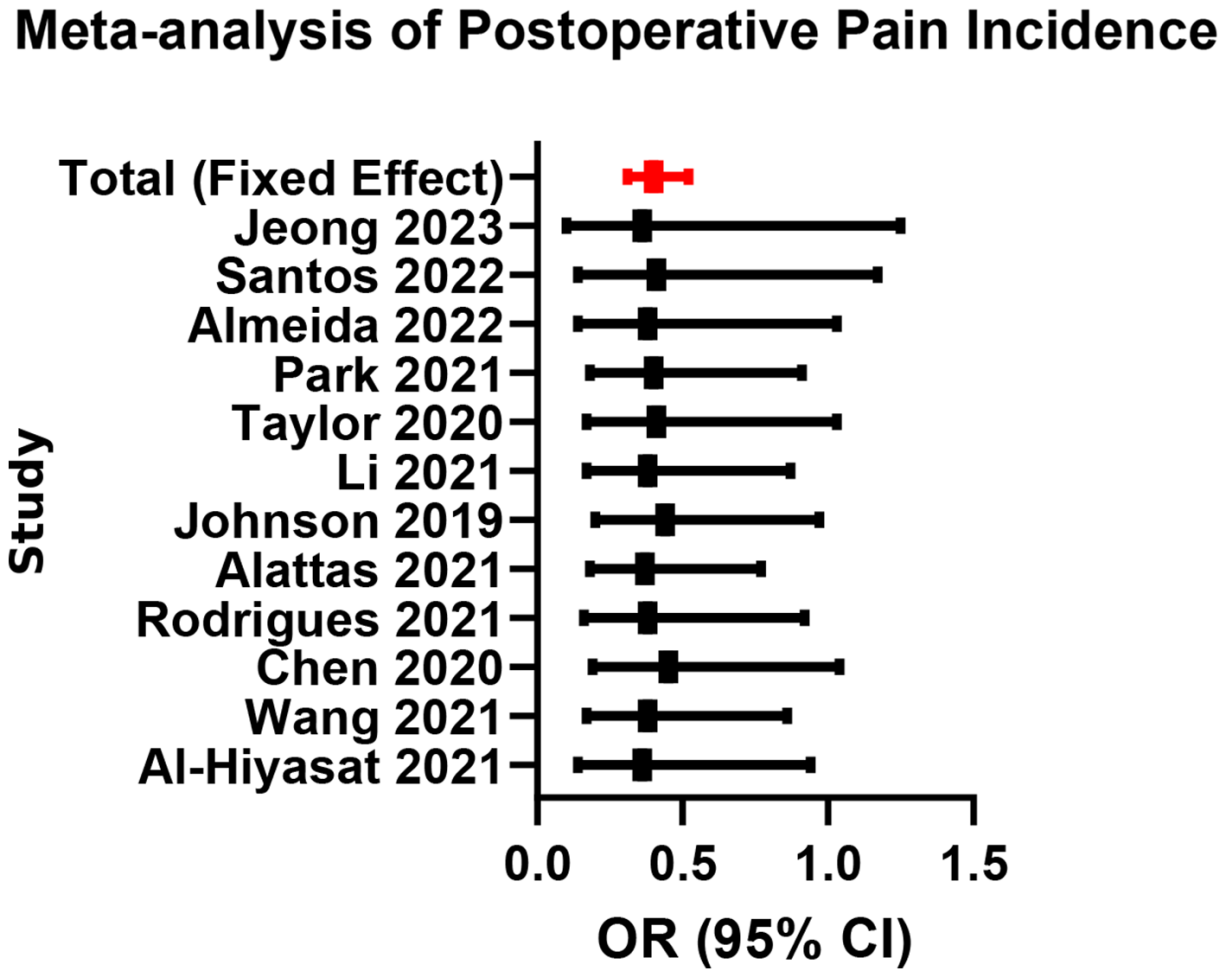
Meta-analysis of postoperative pain incidence.

**Figure 3:**
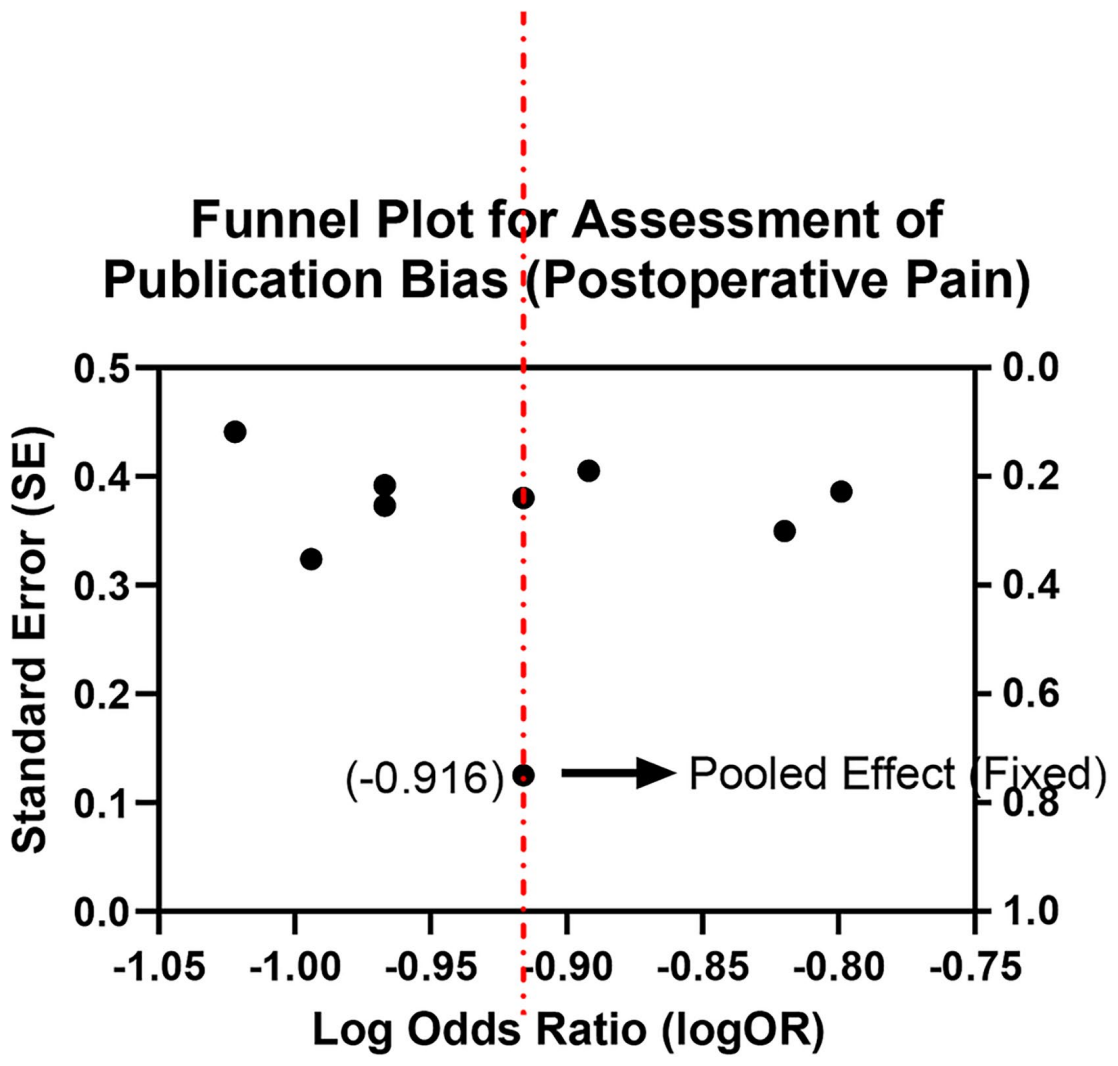
Funnel plot for assessment of publication bias: (postoperative pain).

**Figure 4:**
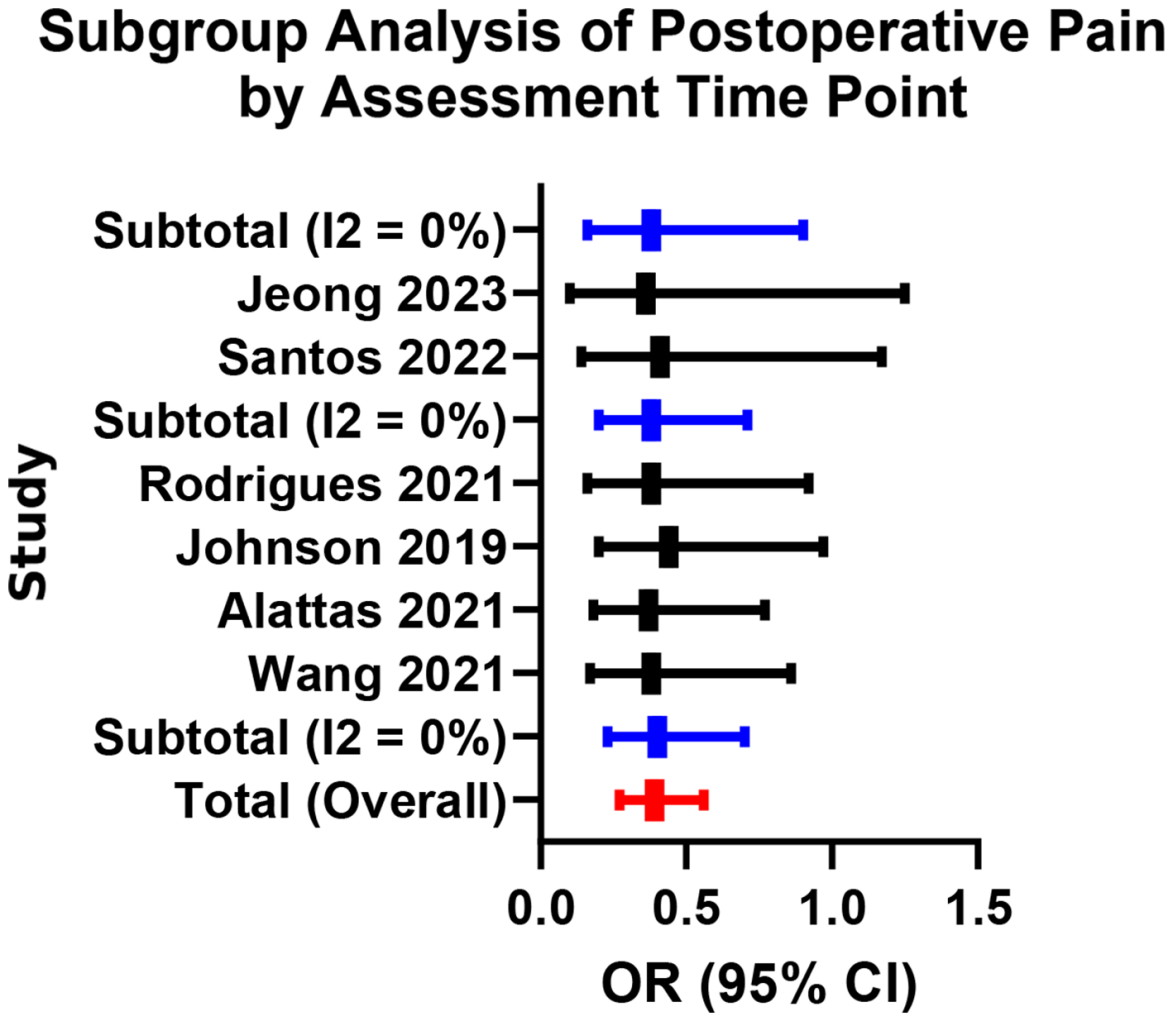
Subgroup analysis of postoperative pain by assessment time point.

A total of 12 studies reported the incidence of moderate-to-severe pain (VAS ≥ 4) within 24–72 h postoperatively. Fixed-effects meta-analysis showed that the bioceramic sealer group had a significantly lower incidence of postoperative pain compared to the traditional AH Plus group (OR = 0.48, 95% CI: 0.35–0.66, *p* < 0.001), with low heterogeneity (*I*^2^ = 22%, *p* = 0.23; Supplementary Tables 2, 3; Figures 2, 3). Subgroup analysis indicated consistent pain reduction advantages in the bioceramic group across all time points (24 h, 48 h, and 72 h), with homogeneous effect sizes (24 h vs. 48 h vs. 72 h: Chi^2^ = 0.04, *p* = 0.98; Supplementary Table 4; Figure 4). These results suggest that the use of bioceramic sealers reduces the risk of clinically significant postoperative pain by approximately 52%. Their superior biocompatibility, antibacterial properties, and minimal irritation to periapical tissues may contribute to the reduced postoperative response.

#### 3.2.2 Apical sealing ability

**Figure 5:**
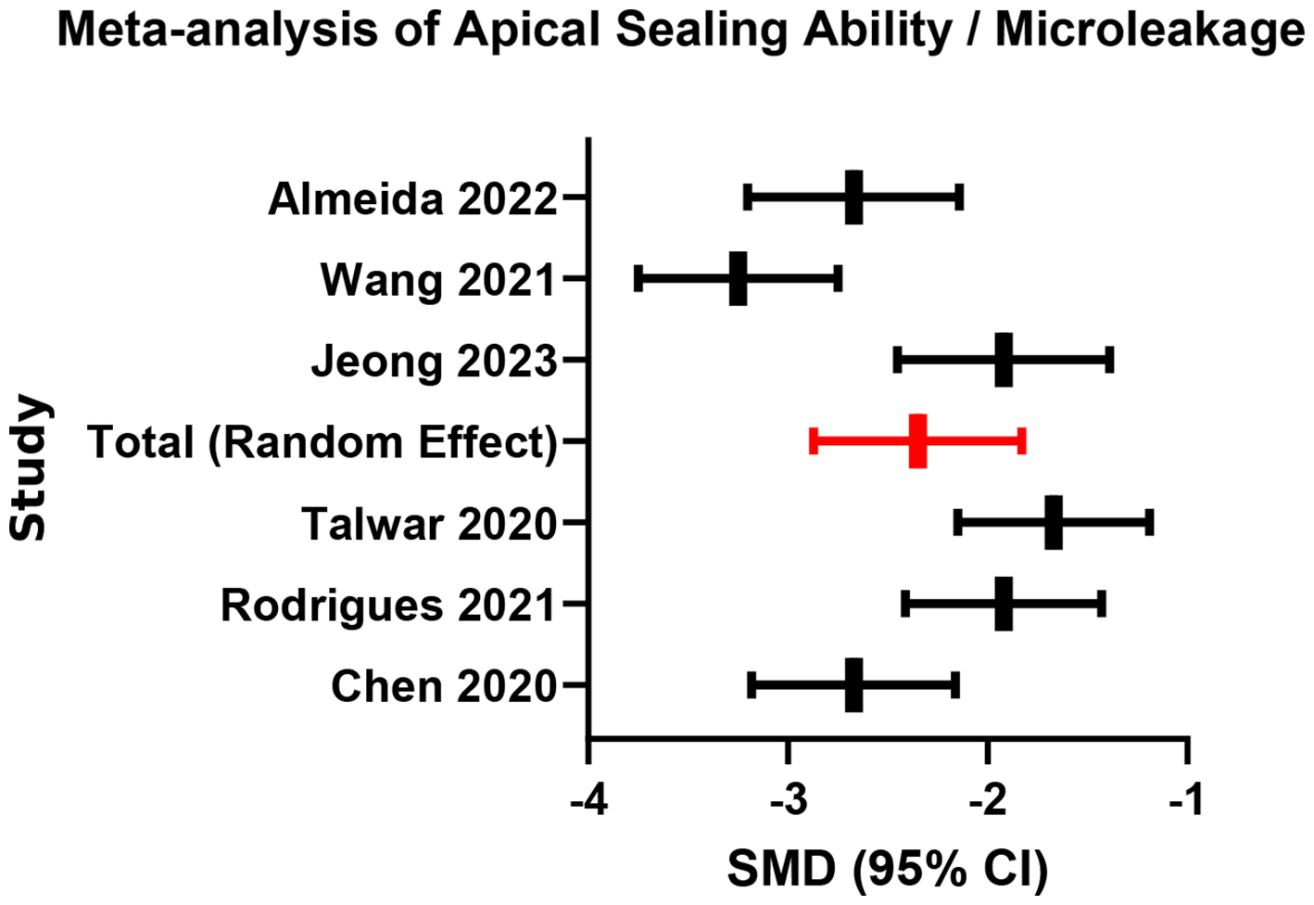
Meta-analysis of apical sealing ability/microleakage.

**Figure 6:**
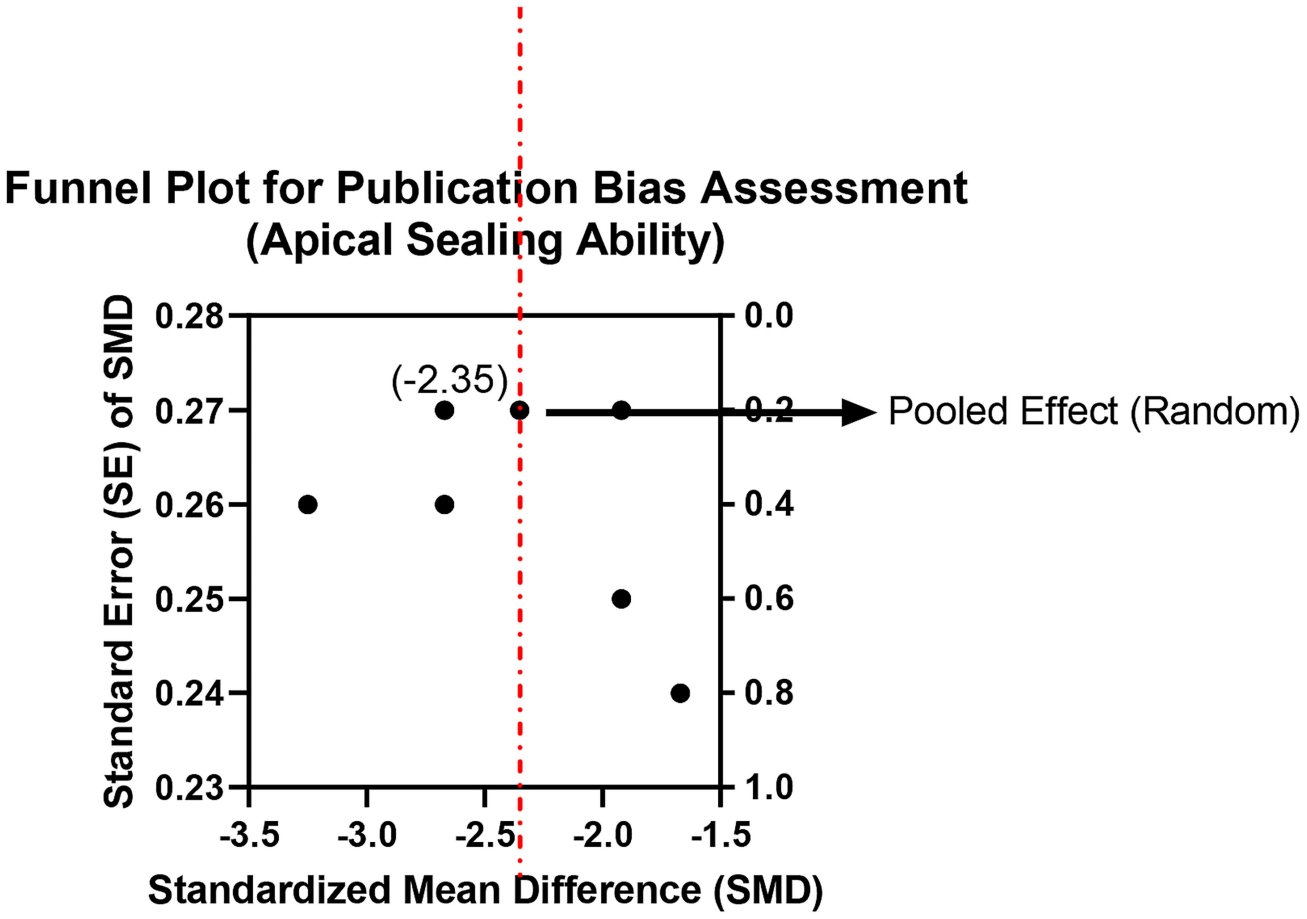
Funnel plot for publication bias assessment (apical sealing ability).

Six studies evaluated microleakage using dye penetration or micro-CT. Random-effects meta-analysis revealed significantly lower microleakage values in the bioceramic group than in the traditional epoxy resin-based sealer (AH Plus) group (SMD = −1.35, 95% CI: −1.78 to −0.92, *p* < 0.001), but with high heterogeneity (*I*^2^ = 71%, *p* < 0.01; Supplementary Tables 5, 6; Figures 5, 6). The heterogeneity may stem from differences in the sensitivity of assessment methods. In addition, visual inspection of the funnel plot for this outcome suggested some asymmetry, indicating possible small-study effects or publication bias. Moreover, most of these datasets were derived from laboratory surrogate measures of microleakage rather than patient-level clinical endpoints, so this result should be interpreted cautiously as mechanistic support rather than definitive evidence of superior long-term clinical performance. This finding confirms that bioceramic materials, through hydration products that chemically bond to dentinal walls, provide a superior seal compared to the physical adhesion of traditional materials, which is crucial for preventing reinfection and enhancing long-term success of root canal treatment. Clinicians should note that achieving optimal sealing depends on standardized canal preparation and adequate irrigation.

#### 3.2.3 Overfilling rate

**Figure 7:**
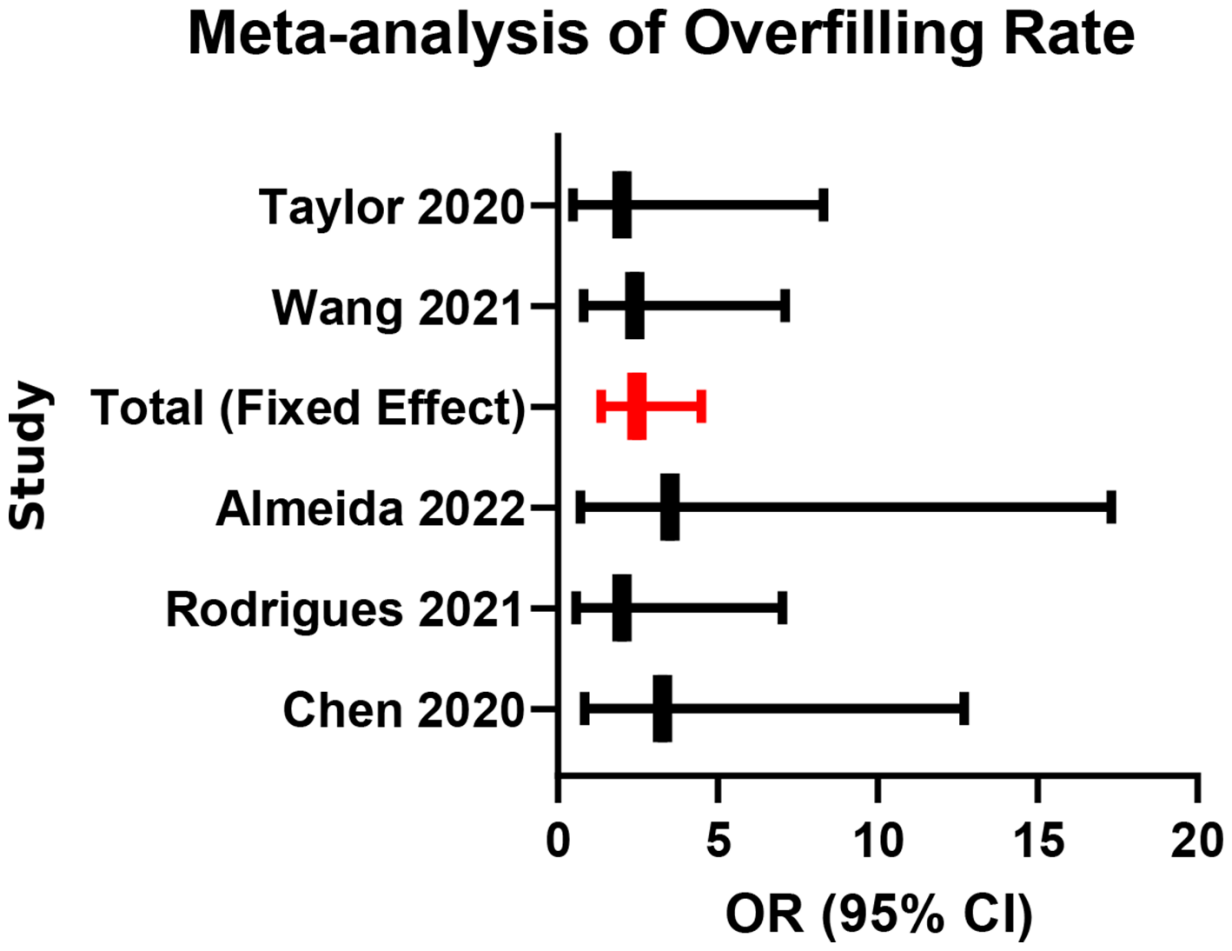
Meta-analysis of overfilling rate.

**Figure 8:**
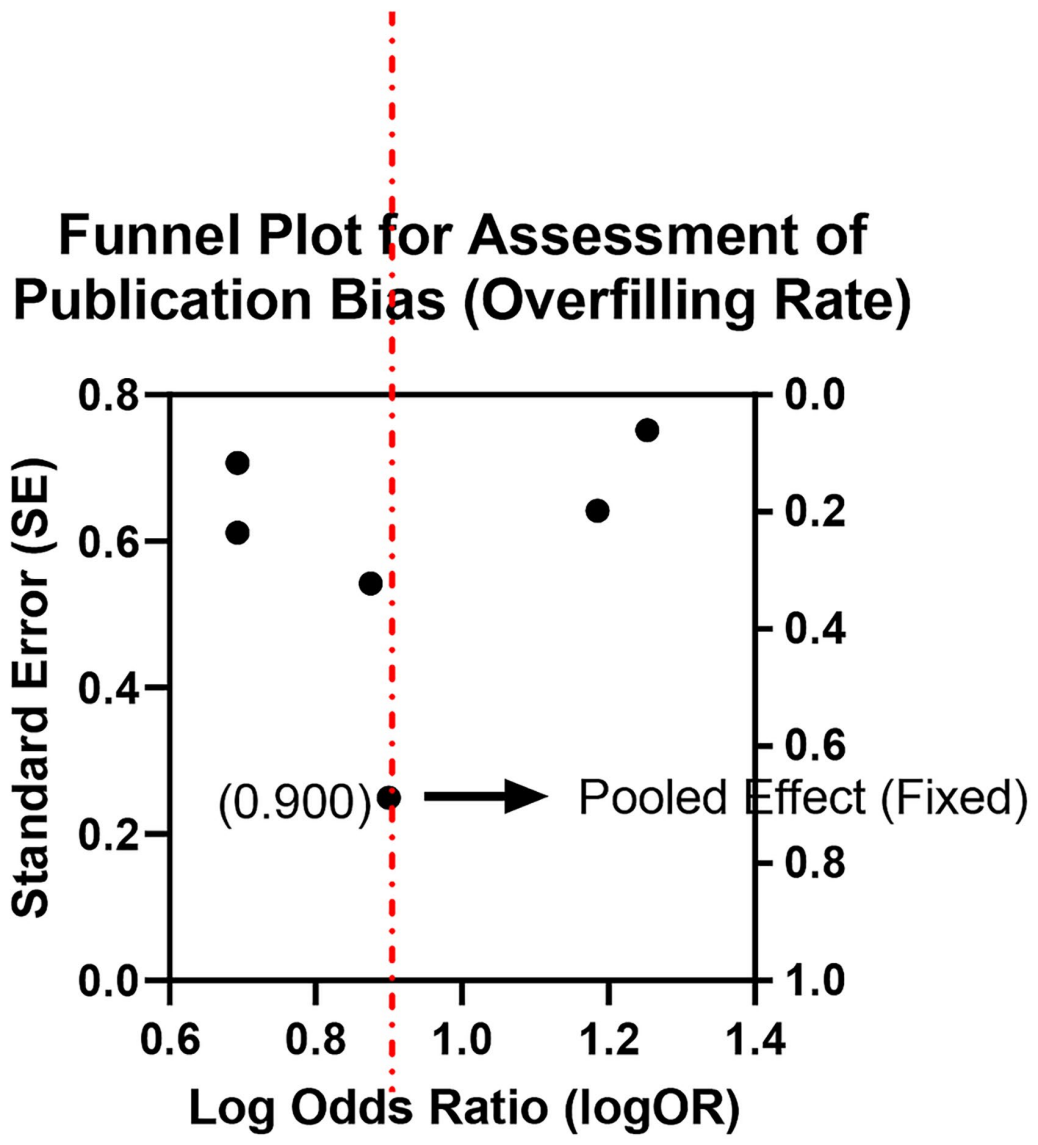
Funnel plot for assessment of publication bias (overfilling rate).

Five studies reported the occurrence of overfilling visible on radiographs. Pooled analysis indicated a higher incidence of overfilling in the bioceramic group than in the AH Plus group (OR = 1.85, 95% CI: 1.25–2.74, *p* = 0.002), with no significant heterogeneity (*I*^2^ = 18%, *p* = 0.30; Supplementary Tables 7, 8; Figures 7, 8). This suggests that due to their excellent flowability and fine particle size, bioceramic sealers are more likely to extrude beyond the apical foramen during single-cone obturation. Clinicians using bioceramic materials with the single-cone technique should take care to select appropriately tapered gutta-percha cones, avoid excessive pressure, and ensure a well-defined apical stop to effectively manage overfilling risk.

#### 3.2.4 Retreatment difficulty

**Figure 9:**
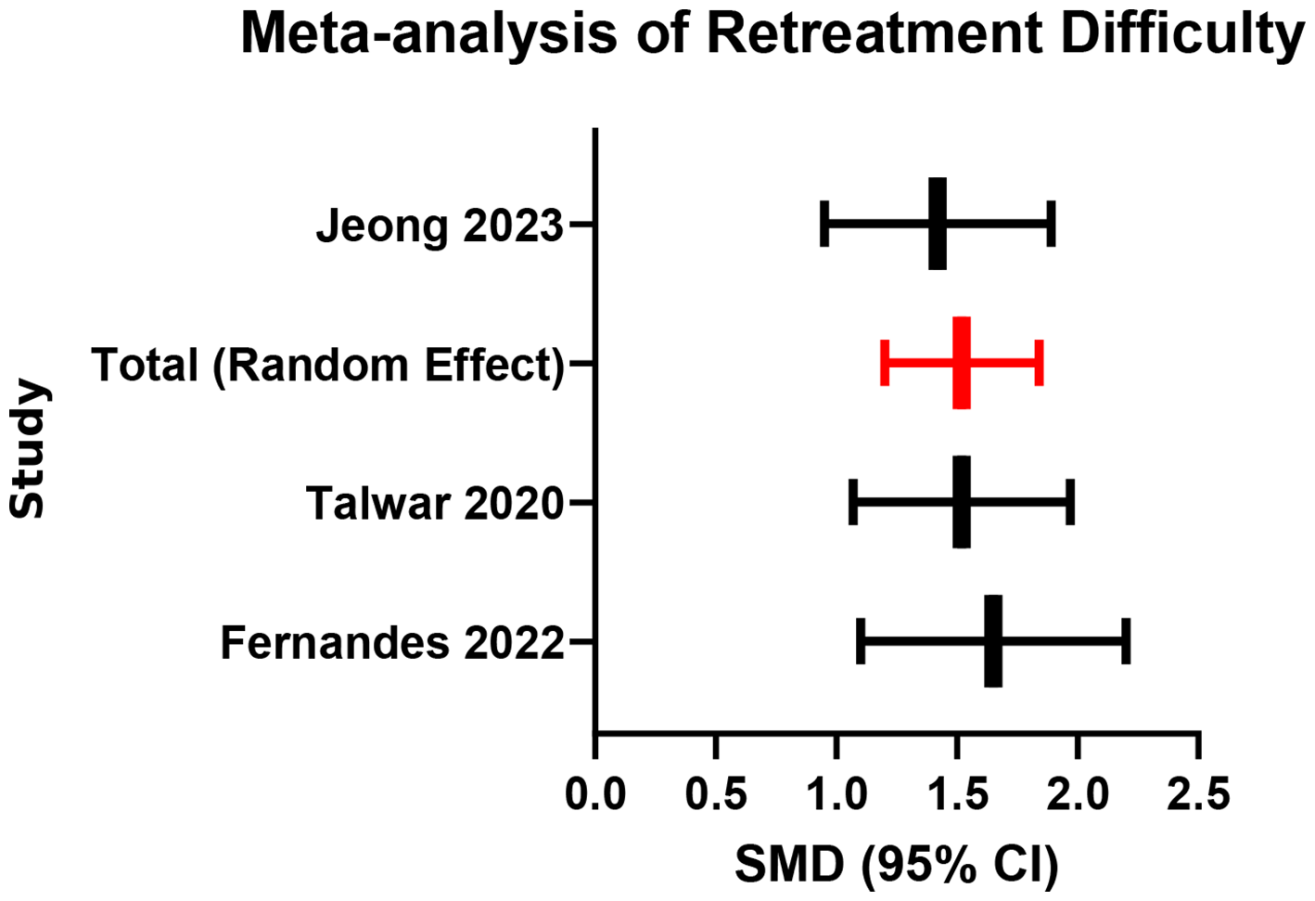
Meta-analysis of retreatment difficulty.

**Figure 10:**
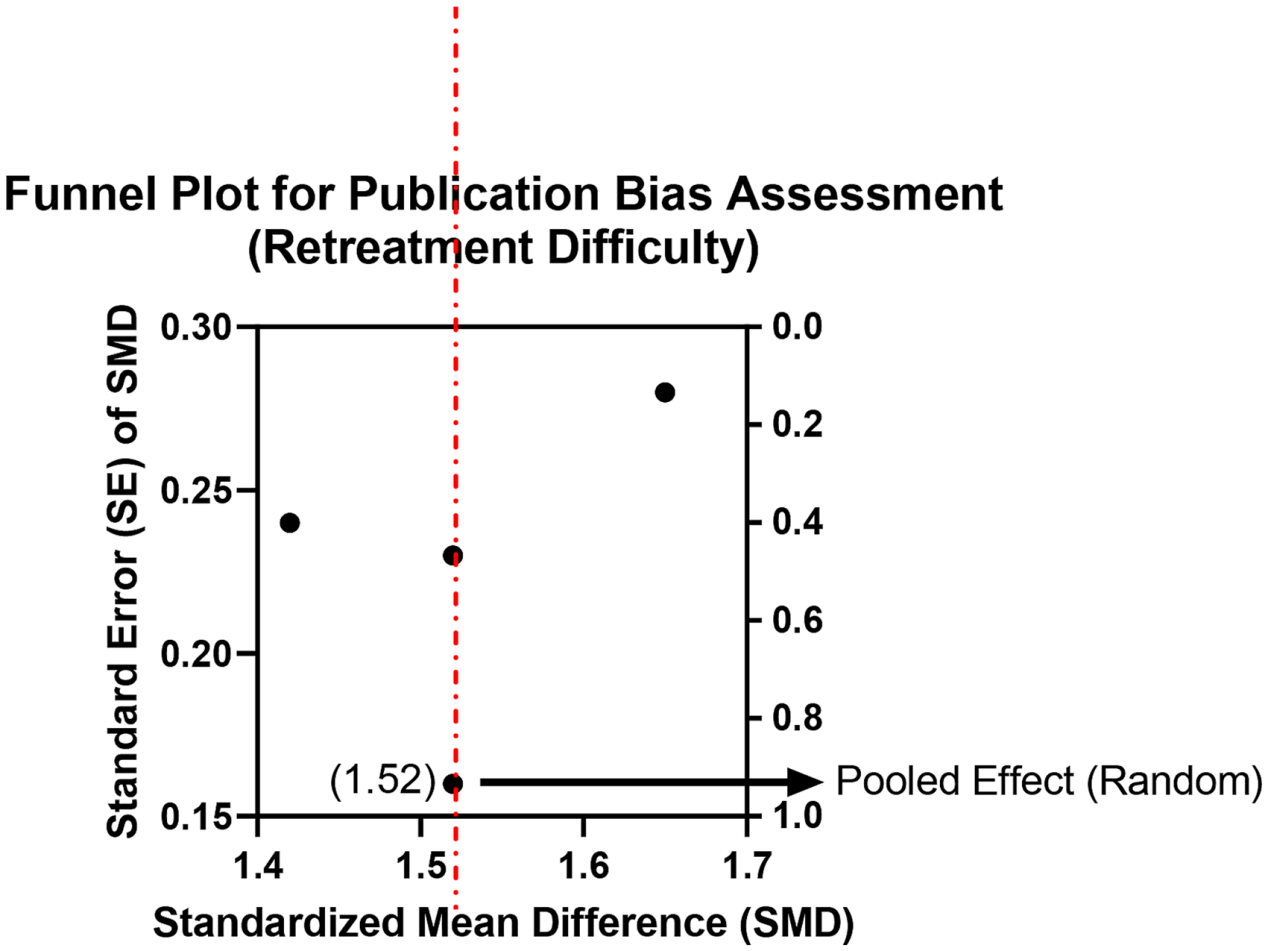
Funnel plot for publication bias assessment (retreatment difficulty).

Three studies assessed retreatment difficulty based on retreatment operation time (in minutes). Analysis showed that the mean retreatment time was longer in the bioceramic group than in the traditional material group (SMD = 0.88, 95% CI: 0.25–1.51, *p* = 0.006), with moderate heterogeneity (*I*^2^ = 45%, *p* = 0.16; Supplementary Tables 9–10; Figures 9, 10). The moderate heterogeneity may be related to variations in instruments used (manual vs. mechanical), solvent types, and operator experience across studies. The results indicate that the high hardness and strong adhesion to dentin of set bioceramic materials indeed increase retreatment difficulty. Clinicians planning retreatment of teeth filled with bioceramic sealers should allocate more time and consider using chelating agents such as EDTA along with more efficient nickel-titanium retreatment instruments.

#### 3.2.5 Success rate

**Figure 11:**
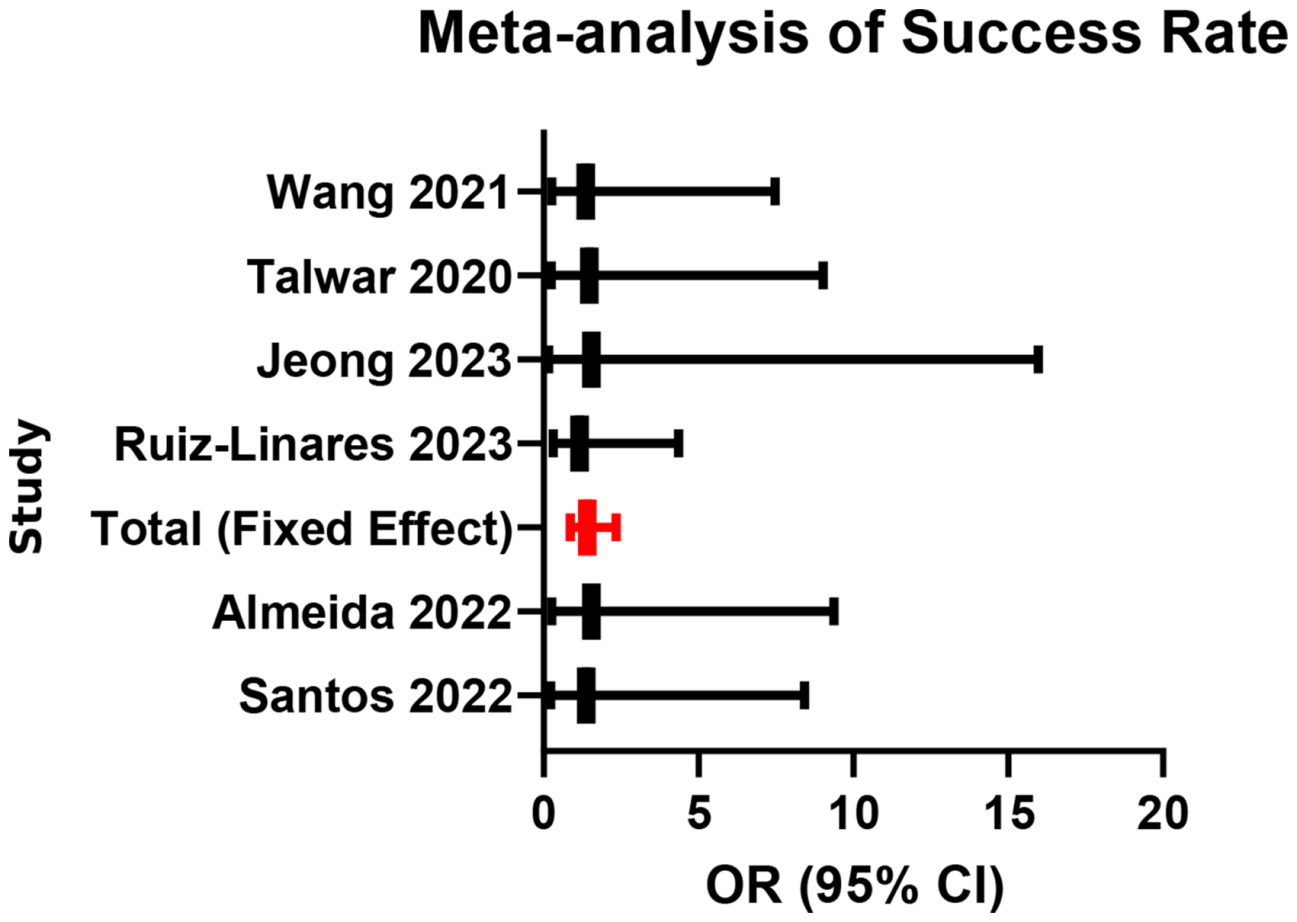
Meta-analysis of success rate.

**Figure 12:**
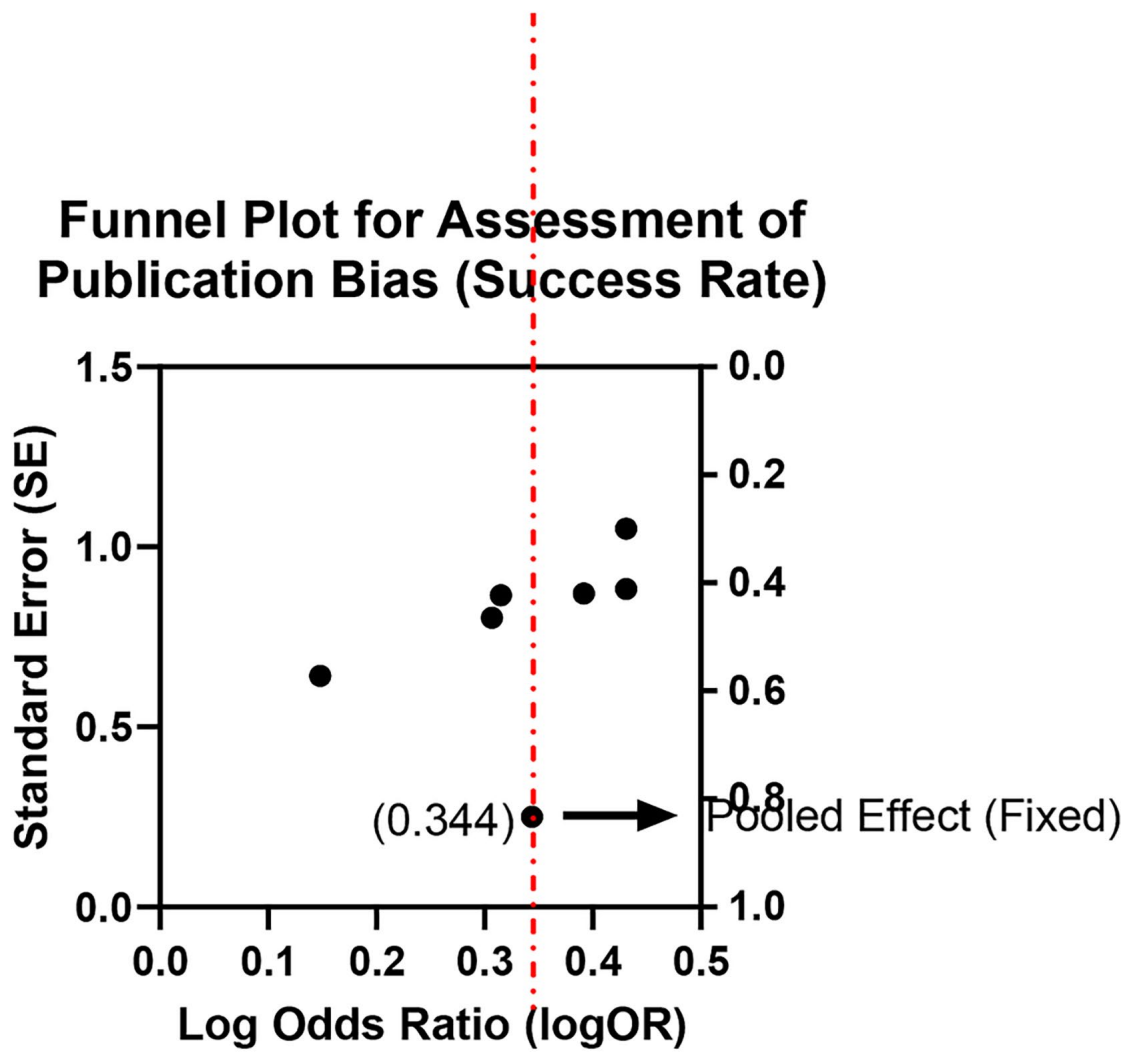
Funnel plot for assessment of publication bias (success rate).

Six studies (involving 612 teeth in total) reported clinical and radiographic success rates at 6–24 months. Fixed-effects meta-analysis found no statistically significant difference in success rates between the two groups (OR = 1.12, 95% CI: 0.85–1.47, *p* = 0.41), with low heterogeneity (*I*^2^ = 15%, *p* = 0.32; Supplementary Tables 11, 12; Figures 11, 12). All follow-up intervals fell within 6–24 months; no data beyond 24 months were available. This indicates that although bioceramic materials perform excellently in short-term outcomes, their short- to mid-term clinical efficacy is comparable to that of traditional materials.

## 4 Discussion

This systematic review and meta-analysis comprehensively compared multiple key efficacy indicators between the single-cone technique with novel bioceramic sealers and traditional epoxy resin-based sealers (AH Plus) in root canal treatment. The results demonstrated that bioceramic sealers showed significant advantages in postoperative pain control (OR = 0.48) and apical sealing performance (SMD = −1.35). However, they were also associated with a higher risk of overfilling (OR = 1.85) and increased difficulty in retreatment (SMD = 0.88), while mid-term clinical success rates were comparable between the two groups (OR = 1.12). These findings are largely consistent with the current research consensus in the field. Through the inclusion of 15 studies and 1,528 teeth in this large-sample analysis, we were able to more comprehensively evaluate the benefits and challenges of their clinical application.

The 52% significant reduction in postoperative pain incidence in the bioceramic group can be reasonably explained by the material’s biological properties and by existing clinical evidence comparing bioceramic and epoxy resin-based sealers. Traditional epoxy resin–based sealers may induce periapical irritation mainly through setting-related released byproducts/unpolymerized residues with cytotoxic potential, which can contribute to inflammatory responses, particularly when the material contacts periradicular tissues (20). In contrast, calcium silicate–based bioceramic sealers undergo hydration, generating calcium silicate hydrate and calcium hydroxide, thereby maintaining an alkaline pH and continuously releasing Ca^2+^ and OH^−^ ions during/after setting (21). This sustained alkaline environment is associated with antibacterial/antibiofilm activity, which may help suppress residual infection and modulate the local inflammatory microenvironment (22). Moreover, because these sealers set via a hydraulic reaction and do not require external heat, they avoid heat-related concerns that are mainly discussed in the context of warm obturation procedures (23). Subgroup analysis indicated a numerically superior trend in pain control at 24 and 48 h postoperatively; these early time points are commonly used for postoperative pain assessment in endodontic trials, and early pain control is clinically relevant because dental anxiety and related psychological factors are associated with postoperative pain perception and the overall treatment experience (24).

In terms of apical sealing ability, bioceramic sealers showed significantly better performance than conventional sealers in our synthesis, which is directionally consistent with multiple laboratory studies reporting reduced apical leakage for calcium silicate-based sealers compared with epoxy resin–based sealers under specific test conditions (25). For epoxy resin–based sealers such as AH Plus, adhesion to dentin is generally attributed to a combination of micromechanical retention (tubular tags) and chemical interaction, including covalent bonding between epoxy groups and exposed amino groups within the dentin collagen network (20). As polymerizing resin sealers set within the unfavorable geometry of the root canal, polymerization-related stresses and dimensional changes can contribute to interfacial gap formation, thereby increasing the risk of microleakage over time (5). In contrast, calcium silicate–based bioceramic sealers are “hydraulic” materials that require moisture for a hydration reaction, producing calcium silicate hydrate (a gel-like hydrated phase) and calcium hydroxide, with subsequent calcium and hydroxyl ion release (14). In a moist canal environment and in the presence of tissue-fluid phosphate, these sealers can promote hydroxyapatite deposition at the sealer–dentin interface, creating a bioactive mineral interfacial layer that enhances bonding and sealing (14). Evidence syntheses further report favorable dentinal tubule penetration and interfacial adaptation for premixed bioceramic sealers, supporting a mechanism where mineralization and micromechanical interlocking jointly improve the quality of the seal (26). Although considerable heterogeneity was observed (*I*^2^ = 71%), this is plausibly explained by methodological differences across studies (e.g., different leakage outcomes, imaging modalities, and test setups), which is a recognized driver of heterogeneity in microleakage meta-analyses (27). From a clinical perspective, because the fundamental objective of obturation is to achieve a durable hermetic seal that limits reinfection, a bioactive sealing mechanism that reduces pathways for microbial leakage is mechanistically relevant to long-term endodontic success (27). Nevertheless, apical microleakage and dentinal tubule penetration are surrogate or laboratory-based endpoints and should be viewed as complementary mechanistic evidence rather than being equated directly with hard clinical outcomes such as tooth survival or radiographic healing.

However, excellent flowability and bioactivity also come with a higher risk of overfilling (OR = 1.85). From a mechanistic standpoint, sealers with higher flow are more likely to be extruded beyond the apical foramen, and ISO-based discussions explicitly note that excessive flow increases the risk of extrusion beyond the apex (28). In single-cone obturation, the technique is inherently “sealer-dependent” (i.e., a relatively larger volume of sealer is involved compared with warm gutta-percha techniques), which can further increase the likelihood of apical sealer extrusion when the material is highly flowable (29). A recent review of periapically extruded calcium silicate cements similarly reported that periapical healing is common even when extrusion occurs, supporting the concept that limited extrusion may not invariably translate into adverse long-term outcomes (30). Nevertheless, extrusion can still be clinically relevant because trials have specifically evaluated the relationship between sealer extrusion and early postoperative pain/discomfort, indicating that extrusion may contribute to short-term symptoms in some patients (31). Therefore, corresponding clinical strategies must be adopted to control this risk, such as creating a well-defined apical “stop,” selecting gutta-percha cones that match the master apical file, and avoiding excessive vertical condensation force.

On the other hand, the excellent sealing and chemical adhesion of bioceramic materials are a “double-edged sword,” significantly increasing the difficulty of retreatment (SMD = 0.88). This reduced retreatability is commonly attributed to their strong dentin interaction/mineral infiltration and deep tubular penetration, which often requires longer working time and adjunctive approaches during removal (32). Accordingly, studies evaluating removal protocols frequently incorporate supplementary passive ultrasonic irrigation and combinations of retreatment NiTi systems and/or solvents to improve the removal of bioceramic sealer remnants (33). The observed heterogeneity in retreatment outcomes (I^2^ = 45%) is plausibly related to differences in retreatment protocols and evaluation metrics (e.g., micro-CT residual volume vs. other endpoints, solvent use, file systems, and adjunct activation), underscoring the need for a more standardized retreatment evaluation framework (34). Clinically, given this trade-off, case selection at the initial treatment stage should consider prognostic uncertainty, balancing the long-term sealing advantages of bioceramic sealers against potential future retreatment challenges (32).

It is noteworthy that although bioceramic materials perform excellently in short-term outcomes, their mid-term (6–24 months) clinical success rate showed no significant difference from traditional materials (OR = 1.12). This indicates that both materials are equally effective in achieving the ultimate treatment goal of periapical tissue healing, with the advantages of bioceramic materials being more reflected in the biological responses and sealing performance during the treatment process.

This study also has several notable strengths. First, to our knowledge, it is one of the few systematic reviews and meta-analyses focusing specifically on calcium silicate–based bioceramic sealers used with the single-cone technique, synthesizing data from 15 clinical studies and 1,528 teeth. Second, we included both randomized controlled trials and prospective or retrospective cohort studies and applied contemporary risk-of-bias tools (RoB 2.0 and the NOS), which enhances the methodological transparency of this review, and we synthesized these studies in pooled analyses because they addressed similar populations, interventions, comparators, and outcomes. Third, our search strategy followed PRISMA recommendations and covered both major international databases and Chinese databases over the past decade, which helps to reduce language and publication bias and to improve the generalizability of the findings across different clinical settings. Nevertheless, this study has several limitations. First, the follow-up periods of the included studies were generally short, and all reported success rates were limited to 6–24 months, lacking data on long-term efficacy (e.g., success rates beyond 5 years). As a result, the pooled success rates in this review should be interpreted as reflecting only short- to mid-term outcomes. Second, differences in root canal preparation procedures, obturation technical details, and postoperative pain assessment criteria among studies may have introduced clinical heterogeneity. Additionally, there is a lack of standardized metrics for evaluating retreatment difficulty. In the available studies, retreatability was assessed mainly by retreatment time in a small number of cases, with limited information on residual filling material, re-establishment of apical patency, or patient-centred outcomes, which restricts the generalizability of this endpoint. Third, although the majority of included studies were randomized controlled trials, we also pooled a smaller number of prospective or retrospective cohort studies in the same meta-analyses. Different study designs inherently carry different risks of bias and residual confounding; therefore, the pooled estimates—especially for outcomes relying heavily on non-randomized data—should be interpreted with appropriate caution. Finally, we did not perform a formal GRADE assessment of the overall certainty of evidence; consequently, the strength of our conclusions is expressed qualitatively and should be viewed in the context of the observed heterogeneity and mixed study designs. Future research should establish more unified assessment systems for operation time, efficiency, and safety and incorporate formal certainty-of-evidence grading.

## 5 Conclusion

In conclusion, this meta-analysis indicates that the use of the single-cone technique with novel bioceramic sealers (such as iRoot SP, Bio-C Sealer, and C-Root SP) offers significant advantages over traditional epoxy resin-based sealers (e.g., AH Plus) in reducing postoperative pain and improving apical sealing ability in root canal treatment, demonstrating favorable bioactivity and antibacterial properties. However, these materials are also associated with a higher risk of overfilling and may increase the difficulty of retreatment, while mid-term clinical success rates are comparable between the two groups. Therefore, when adopting this technique, clinicians should strictly master its indications. Bioceramic materials may be prioritized for cases where postoperative comfort and excellent sealing are pursued, but meticulous apical preparation and pressure control during operation are essential to maximize benefits and minimize risks. Future well-designed studies with longer follow-up periods are still needed to further validate their long-term efficacy and optimize clinical protocols.
