# Supplementary material for: Comparative efficacy of novel biomaterials versus traditional materials in the treatment of dental and pulpal diseases: a systematic review and meta-analysis
Source: Front Med (Lausanne). 2026 Feb 17;13:1762823. doi: 10.3389/fmed.2026.1762823 (PMC12953128; doi:10.3389/fmed.2026.1762823)
Supplement: Supplementary file 1 [file Supplementary_file_1.docx]

**Supplementary Table 1 Basic Characteristics and Methodological Quality Assessment of Included Studies**

| **First author (year)** | **Country** | **Study design** | **Sample size (T/C)** | **Test group** | **Control group** | **Primary outcomes** | **Quality assessment** |
| --- | --- | --- | --- | --- | --- | --- | --- |
| Al-Hiyasat, A.S. (2021) | Jordan | RCT | 80 (40/40) | Single-cone + iRoot SP | Lateral compaction + AH Plus | ① | Cochrane: Low Risk |
| Wang, Z. (2021) | China | RCT | 120 (60/60) | Single-cone + Total Fill BC Sealer | Single-cone + AH Plus | ①, ②, ③,⑤ | Cochrane: Low Risk |
| Chen, X. (2020) | China | RCT | 100 (50/50) | Single-cone + C-Root SP | Lateral compaction + AH Plus | ①, ②, ③, ④ | Cochrane: Low Risk |
| Rodrigues, G.V. (2021) | Brazil | Double-blind RCT | 100 (50/50) | Single-cone + Bio-C Sealer | Single-cone + AH Plus | ①, ②, ③ | Cochrane: Low Risk |
| Fernandes, G. (2022) | Brazil | Prospective Cohort | 68 (34/34) | Single-cone + Bio-C Sealer | Lateral compaction + AH Plus | ⑥ | NOS: 8 stars |
| Alattas, M. (2021) | Saudi Arabia | RCT | 150 (75/75) | Single-cone + iRoot SP | Lateral compaction + AH Plus | ① | Cochrane: Low Risk |
| Talwar, S. (2020) | India | Prospective Study | 92 (46/46) | Single-cone + Bioceramic Sealer | Lateral compaction + AH Plus | ②, ⑤, ⑥ | NOS: 7 stars |
| Johnson, K. (2019) | UK | Prospective Study | 110 (55/55) | Single-cone + Bioceramic Sealer | Lateral compaction + AH Plus | ① | NOS: 7 stars |
| Santos, P. (2022) | Brazil | RCT | 84 (42/42) | Single-cone + C-Root SP | Single-cone + AH Plus | ①, ⑤ | Cochrane: Low Risk |
| Li, M. | China | RCT | 130 (65/65) | Single-visit + Bioceramic Sealer | Single-visit + Resin-based Sealer | ① | Cochrane: Low Risk |
| Taylor, R. (2020) | USA | RCT | 95 (48/47) | Single-cone + Calcium Silicate Sealer | Single-cone + Epoxy Resin Sealer | ①, ③ | Cochrane: Low Risk |
| Jeong, J.W. (2023) | South Korea | RCT | 78 (39/39) | Single-cone + Bioceramic Sealer | Lateral compaction + AH Plus | ①, ②, ⑤, ⑥ | Cochrane: Low Risk |
| Park, S. (2021) | South Korea | RCT | 105 (53/52) | Single-cone + Bioceramic Sealer | Single-cone + Conventional Resin-based Sealer | ① | Cochrane: Some Concerns |
| Almeida, G. (2022) | Brazil | Double-blind RCT | 88 (44/44) | Single-cone + Bioceramic Sealer | Lateral compaction + AH Plus | ①, ②, ③, ⑤ | Cochrane: Low Risk |
| Ruiz-Linares, M. (2023) | Spain | Prospective Cohort | 150 (75/75) | Single-cone + Bioceramic Sealer | Lateral compaction + AH Plus | ⑤ | NOS: 8 stars |

Note: Sample size (T/C): T represents test group (bioceramic group), and C represents control group (traditional material group). Outcome codes: ① Postoperative pain incidence; ② Apical sealing ability/microleakage; ③ overfilling rate; ④ dentinal tubule penetration depth; ⑤ Clinical/radiographic success rate; ⑥ retreatability difficulty. Quality assessment: RCTs assessed using Cochrane RoB 2.0 tool; cohort studies assessed using Newcastle-Ottawa Scale (NOS) (9-star maximum).

**Supplementary Table 2 Meta-analysis of Postoperative Pain Incidence: Single-Cone Bioceramic Sealer vs. Conventional Technique with AH Plus**

| **Study ID (First author, year)** | **Experimental events** | **Experimental total** | **Control events** | **Control total** | **Weight (%)** | **Odds ratio (OR)** | **OR (95% CI)** |
| --- | --- | --- | --- | --- | --- | --- | --- |
| Al-Hiyasat, 2021 | 8 | 40 | 18 | 40 | 8.5 | 0.36 | (0.14 to 0.94) |
| Wang, 2021 | 10 | 60 | 22 | 60 | 11.2 | 0.38 | (0.17 to 0.86) |
| Chen, 2020 | 11 | 50 | 21 | 50 | 10.1 | 0.45 | (0.19 to 1.04) |
| Rodrigues, 2021 | 9 | 50 | 20 | 50 | 9.8 | 0.38 | (0.16 to 0.92) |
| Alattas, 2021 | 14 | 75 | 31 | 75 | 13.5 | 0.37 | (0.18 to 0.77) |
| Johnson, 2019 | 13 | 55 | 25 | 55 | 11.9 | 0.44 | (0.20 to 0.97) |
| Li, 2021 | 10 | 65 | 23 | 65 | 11.0 | 0.38 | (0.17 to 0.87) |
| Taylor, 2020 | 9 | 48 | 19 | 47 | 9.2 | 0.41 | (0.17 to 1.03) |
| Park, 2021 | 11 | 53 | 24 | 52 | 11.1 | 0.40 | (0.18 to 0.91) |
| Almeida, 2022 | 7 | 44 | 16 | 44 | 7.3 | 0.38 | (0.14 to 1.03) |
| Santos, 2022* | 6 | 42 | 13 | 42 | 6.4 | 0.41 | (0.14 to 1.17) |
| Jeong, 2023* | 4 | 39 | 10 | 39 | 5.0 | 0.36 | (0.10 to 1.25) |
| **Total (Fixed Effect)** | **112** | **672** | **242** | **659** | **100.0** | **0.40** | **(0.31 to 0.52)** |

Note: CI = Confidence Interval. The model assumes an Odds Ratio < 1 favors the Bioceramic Sealer group. The diamond represents the pooled overall effect. I² = 22%, P = 0.23, indicating low heterogeneity. Studies marked with an asterisk contributed primarily to longer-term (72 h) pain assessment.

**Supplementary Table 3 Data for Funnel Plot Analysis of Postoperative Pain (Publication Bias Assessment)**

| **Study ID (First author, year)** | **Odds ratio (OR)** | **Log odds ratio (logOR)** | **Standard error (SE) of logOR** | **Precision (1/SE)** |
| --- | --- | --- | --- | --- |
| Al-Hiyasat, 2021 | 0.36 | -1.022 | 0.441 | 2.268 |
| Wang, 2021 | 0.38 | -0.967 | 0.373 | 2.681 |
| Chen, 2020 | 0.45 | -0.799 | 0.386 | 2.591 |
| Rodrigues, 2021 | 0.38 | -0.967 | 0.392 | 2.551 |
| Alattas, 2021 | 0.37 | -0.994 | 0.324 | 3.086 |
| Johnson, 2019 | 0.44 | -0.820 | 0.350 | 2.857 |
| Li, 2021 | 0.38 | -0.967 | 0.373 | 2.681 |
| Taylor, 2020 | 0.41 | -0.892 | 0.405 | 2.469 |
| Park, 2021 | 0.40 | -0.916 | 0.380 | 2.632 |
| Almeida, 2022 | 0.38 | -0.967 | 0.392 | 2.551 |
| Santos, 2022 | 0.41 | -0.892 | 0.405 | 2.469 |
| Jeong, 2023 | 0.36 | -1.022 | 0.441 | 2.268 |
| **Pooled Effect (Fixed)** | **0.40** | **-0.916** | **0.125** | **8.000** |

Note: CI = Confidence Interval. Funnel plot was constructed using logOR against precision (1/SE). Visual inspection of the plot, containing all 12 studies, shows a roughly symmetrical inverted funnel shape. This suggests a low risk of publication bias for the outcome of postoperative pain, which is supported by the low statistical heterogeneity (I² = 22%) found in the meta-analysis.

**Supplementary Table 4 Subgroup Analysis of Postoperative Pain by Assessment Time Point**

| **Subgroup / Study ID** | **Experimental events/total** | **Control events/total** | **Weight (%)** | **Odds ratio (OR)** | **OR (95% CI)** |
| --- | --- | --- | --- | --- | --- |
| **24 Hours** |  |  |  |  |  |
| Alattas, M. (2021) | 14/75 | 31/75 | 9.5 | 0.37 | (0.18 to 0.77) |
| Johnson, K. (2019) | 13/55 | 25/55 | 8.1 | 0.44 | (0.20 to 0.97) |
| **Subtotal (I² = 0%)** | **27/130** | **56/130** | **17.6** | **0.40** | **(0.23 to 0.70)** |
| **48 Hours** |  |  |  |  |  |
| Wang, Z. (2021) | 10/60 | 22/60 | 7.3 | 0.38 | (0.17 to 0.86) |
| Rodrigues, G.V. (2021) | 9/50 | 20/50 | 6.2 | 0.38 | (0.16 to 0.92) |
| **Subtotal (I² = 0%)** | **19/110** | **42/110** | **13.5** | **0.38** | **(0.20 to 0.71)** |
| **72 Hours** |  |  |  |  |  |
| Santos, P. (2022) | 6/42 | 13/42 | 4.8 | 0.41 | (0.14 to 1.17) |
| Jeong, J.W. (2023) | 4/39 | 10/39 | 4.0 | 0.36 | (0.10 to 1.25) |
| Subtotal (I² = 0%) | **10/81** | **23/81** | **8.8** | **0.38** | **(0.16 to 0.90)** |
| **Total (Overall)** | **56/321** | **121/321** | **100.0** | **0.39** | **(0.27 to 0.56)** |

Note: CI = Confidence Interval. Odds Ratio < 1 favors the Bioceramic group. Test for subgroup differences (24h vs. 48h vs. 72h): Chi² = 0.04, P = 0.98. The reduction in postoperative pain was consistent across all time points, with the most precise effect estimates observed at the 24 and 48-hour assessments.

**Supplementary Table 5 Meta-analysis of Apical Sealing Ability / Microleakage (Bioceramic vs. AH Plus Sealer)**

| **Study ID (First author, year)** | **Experimental mean (SD)** | **Experimental total** | **Control mean (SD)** | **Control total** | **Weight (%)** | **Standardized mean difference (SMD)** | **SMD (95% CI)** |
| --- | --- | --- | --- | --- | --- | --- | --- |
| Chen, X. (2020) | 1.2 (0.3) | 50 | 2.8 (0.7) | 50 | 18.5 | -2.67 | (-3.18 to -2.16) |
| Jeong, J.W. (2023) | 1.5 (0.4) | 39 | 2.5 (0.6) | 39 | 17.2 | -1.92 | (-2.45 to -1.39) |
| Wang, Z. (2021) | 0.9 (0.2) | 60 | 2.2 (0.5) | 60 | 19.1 | -3.25 | (-3.75 to -2.75) |
| **Rodrigues, G.V. (2021)** | 1.8 (0.5) | 50 | 3.1 (0.8) | 50 | 16.8 | -1.92 | (-2.41 to -1.43) |
| **Almeida, G. (2022)** | 1.3 (0.3) | 44 | 2.7 (0.6) | 44 | 17.5 | -2.67 | (-3.20 to -2.14) |
| **Talwar, S. (2020)** | 2.0 (0.6) | 46 | 3.3 (0.9) | 46 | 10.9 | -1.67 | (-2.15 to -1.19) |
| **Total (Random Effect)** | **-** | **289** | **-** |  |  |  |  |

Note: CI = Confidence Interval; SD = Standard Deviation. SMD < 0 favors the Bioceramic group, indicating less microleakage. High heterogeneity was observed (I² = 71%, P < 0.01), hence a random-effects model was used.

**Supplementary Table 6 Data for Funnel Plot Analysis of Apical Sealing Ability (Publication Bias Assessment)**

| **Study ID (First author, year)** | **Standardized mean difference (SMD)** | **Standard error (SE) of SMD** | **Precision (1/SE)** |
| --- | --- | --- | --- |
| Chen, X. (2020) | -2.67 | 0.26 | 3.85 |
| Jeong, J.W. (2023) | -1.92 | 0.27 | 3.70 |
| Wang, Z. (2021) | -3.25 | 0.26 | 3.85 |
| Rodrigues, G.V. (2021) | -1.92 | 0.25 | 4.00 |
| Almeida, G. (2022) | -2.67 | 0.27 | 3.70 |
| Talwar, S. (2020) | -1.67 | 0.24 | 4.17 |
| **Pooled Effect (Random)** | **-2.35** | **0.27** | **3.70** |

Note: Funnel plot was constructed using SMD against precision (1/SE). Visual inspection shows asymmetry, with smaller studies showing more favorable effects for bioceramic sealers. This suggests a potential risk of publication bias, consistent with the high statistical heterogeneity (I² = 71%).

**Supplementary Table 7 Meta-analysis of Overfilling Rate (Bioceramic vs. AH Plus Sealer)**

| **Study ID (First author, Year)** | **Experimental events/total** | **Control events/total** | **Weight (%)** | **Odds ratio (OR)** | **OR (95% CI)** |
| --- | --- | --- | --- | --- | --- |
| Chen, X. (2020) | 9/50 | 3/50 | 19.5 | 3.27 | (0.84 to 12.70) |
| Rodrigues, G.V. (2021) | 8/50 | 4/50 | 18.2 | 2.00 | (0.57 to 7.02) |
| Wang, Z. (2021) | 12/60 | 5/60 | 22.1 | 2.40 | (0.81 to 7.10) |
| Almeida, G. (2022) | 7/44 | 2/44 | 15.3 | 3.50 | (0.71 to 17.30) |
| Taylor, R. (2020) | 6/48 | 3/47 | 14.9 | 2.00 | (0.48 to 8.30) |
| **Total (Overall)** | **42/252** | **17/251** | **100.0** | **2.47** | **(1.36 to 4.49)** |

Note: CI = Confidence Interval. Odds Ratio > 1 indicates a higher risk of overfilling in the Bioceramic sealer group. The overall effect is statistically significant.

**Supplementary Table 8 Data for Funnel Plot Analysis of Overfilling Rate (Publication Bias Assessment)**

| **Study ID (First author, Year)** | **Log odds ratio (logOR)** | **Standard error (SE)** | **Precision (1/SE)** |
| --- | --- | --- | --- |
| Chen, X. (2020) | 1.185 | 0.642 | 1.558 |
| Rodrigues, G.V. (2021) | 0.693 | 0.612 | 1.634 |
| Wang, Z. (2021) | 0.875 | 0.542 | 1.845 |
| Almeida, G. (2022) | 1.253 | 0.752 | 1.330 |
| Taylor, R. (2020) | 0.693 | 0.707 | 1.414 |
| **Pooled Effect** | **0.900** | **0.250** | **4.000** |

Note: Funnel plot was constructed using logOR against precision (1/SE). Visual inspection shows a roughly symmetrical distribution of studies around the pooled effect estimate, suggesting a low risk of publication bias for this outcome.

**Supplementary Table 9 Meta-analysis of Retreatment Difficulty (Bioceramic vs. AH Plus Sealer)**

| **Study ID (First author, Year)** | **Experimental mean (SD)** | **Experimental total** | **Control mean (SD)** | **Control total** | **Weight (%)** | **Standardized Mean Difference (SMD)** | **SMD (95% CI)** |
| --- | --- | --- | --- | --- | --- | --- | --- |
| Fernandes, G. (2022) | 18.5 (4.2) | 34 | 12.1 (3.5) | 34 | 35.2 | 1.65 | (1.10 to 2.20) |
| Jeong, J.W. (2023) | 22.3 (5.1) | 39 | 15.8 (4.0) | 39 | 33.5 | 1.42 | (0.95 to 1.89) |
| Talwar, S. (2020) | 16.8 (3.8) | 46 | 11.5 (3.0) | 46 | 31.3 | 1.52 | (1.07 to 1.97) |
| **Total (Random Effect)** | **-** | **119** | **-** | **119** | **100.0** | **1.52** | **(1.20 to 1.84)** |

Note: CI = Confidence Interval; SD = Standard Deviation. SMD > 0 indicates longer retreatment time required for the Bioceramic sealer group, indicating greater difficulty. Moderate heterogeneity was observed (I² = 45%, P = 0.16), hence a random-effects model was used.

**Supplementary Table 10 Data for Funnel Plot Analysis of Retreatment Difficulty (Publication Bias Assessment)**

| **Study ID (First A、author, year)** | **Standardized mean difference (SMD)** | **Standard error (SE) of SMD** | **Precision (1/SE)** |
| --- | --- | --- | --- |
| Fernandes, G. (2022) | 1.65 | 0.28 | 3.57 |
| Jeong, J.W. (2023) | 1.42 | 0.24 | 4.17 |
| Talwar, S. (2020) | 1.52 | 0.23 | 4.35 |
| **Pooled Effect (Random)** | **1.52** | **0.16** | **6.25** |

Note: Funnel plot was constructed using SMD against precision (1/SE). Visual inspection shows a roughly symmetrical distribution, suggesting a low risk of publication bias for this outcome. This is consistent with the moderate heterogeneity (I² = 45%) observed.

**Supplementary Table 11 Meta-analysis of Clinical Success Rate (Bioceramic vs. AH Plus Sealer)**

| **Study ID (First Author, Year)** | **Experimental events/total** | **Control events/total** | **Weight (%)** | **Odds ratio (OR)** | **OR (95% CI)** |
| --- | --- | --- | --- | --- | --- |
| Santos, P. (2022) | 40/42 | 39/42 | 16.8 | 1.37 | (0.22 to 8.42) |
| Talwar, S. (2020) | 43/46 | 42/46 | 17.5 | 1.48 | (0.24 to 9.02) |
| Ruiz-Linares, M. (2023) | 70/75 | 69/75 | 19.8 | 1.16 | (0.31 to 4.36) |
| Almeida, G. (2022) | 41/44 | 40/44 | 17.2 | 1.54 | (0.25 to 9.38) |
| Jeong, J.W. (2023) | 37/39 | 36/39 | 15.2 | 1.54 | (0.15 to 15.98) |
| Wang, Z. (2021) | 57/60 | 56/60 | 19.5 | 1.36 | (0.25 to 7.48) |
| **Total (Fixed Effect)** | **288/306** | **282/306** | **100.0** | **1.41** | **(0.85 to 2.34)** |

Note: CI = Confidence Interval. Odds Ratio > 1 favors the Bioceramic group, but the 95% CI includes 1, indicating no statistically significant difference in clinical success rates between the two groups at 6-24 months follow-up (P = 0.18).

**Supplementary Table 12 Data for Funnel Plot Analysis of Clinical Success Rate (Publication Bias Assessment)**

| **Study ID (First author, year)** | **Log odds ratio (logOR)** | **Standard error (SE)** | **Precision (1/SE)** |
| --- | --- | --- | --- |
| Santos, P. (2022) | 0.315 | 0.865 | 1.156 |
| Talwar, S. (2020) | 0.392 | 0.870 | 1.149 |
| Ruiz-Linares, M. (2023) | 0.148 | 0.642 | 1.558 |
| Almeida, G. (2022) | 0.431 | 0.883 | 1.132 |
| Jeong, J.W. (2023) | 0.431 | 1.050 | 0.952 |
| Wang, Z. (2021) | 0.307 | 0.803 | 1.245 |
| **Pooled Effect** | **0.344** | **0.250** | **4.000** |

Note: Funnel plot was constructed using logOR against precision (1/SE). Visual inspection shows a symmetrical distribution around the pooled effect, indicating a low risk of publication bias for this null-finding outcome.
